# Supplementary material for: High-resolution X-ray imaging via spatially decoupled heavy-atom antennas in organic scintillators
Source: Nat Commun. 2026 Feb 19;17:2949. doi: 10.1038/s41467-026-69795-1 (PMC13031387; doi:10.1038/s41467-026-69795-1)
Supplement: Supplementary file 1 — Supplementary Information [file 41467_2026_69795_MOESM1_ESM.pdf]

High-Resolution X-Ray Imaging via Spatially Decoupled Heavy-Atom Antennas in Organic Scintillators

Chensen Li[1*†], Yaohui Li[2†], Minghui Wu[3], Fan-Cheng Kong[4], Binxia Jia[2], Zonghang Liu[5], Xilong Wei[5], Philip C.Y. Chow[4], Zhicheng Wang[6], Xiaoming Li[6], Bo Xu[1*], Zheng Zhao[5], Ryan T. K. Kwok[7], Jacky W. Y. Lam[7*], Yucheng Liu[2*], Shengzhong (Frank) Liu[2], and Ben Zhong Tang[5*]

Synthetic routes

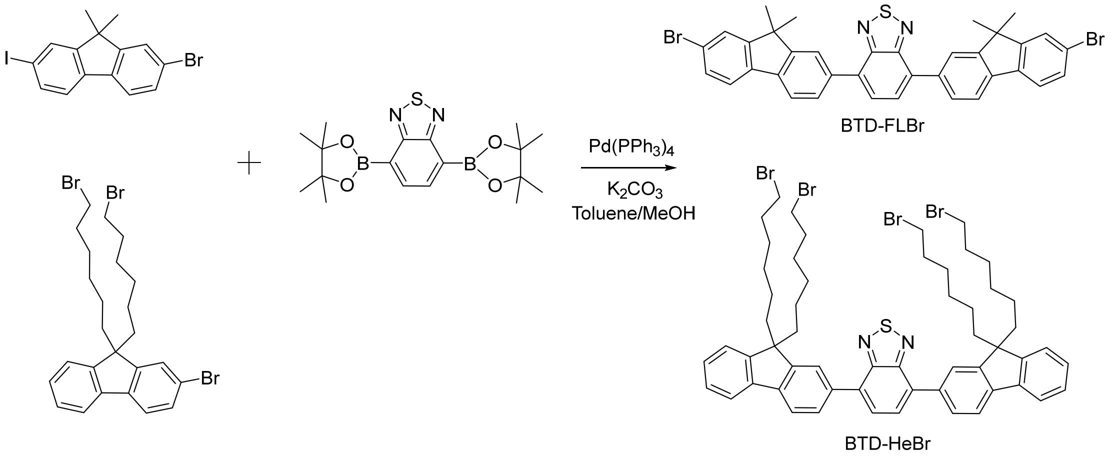

Supplementary Fig. 1. The synthetic routes of BTD-FLBr, and BTD-HeBr.

Synthesis of BTD-FLBr: 4,7-Bis(4,4,5,5-tetramethyl-1,3,2-dioxaborolan-2-yl)benzo[c][1,2,5]thiadiazole (388 mg 1 mmol), tetrakis-(triphenylphosphine)palladium(0) (16 mg, 0.0144 mol), 2 M K_2CO_3 (6 mL) and toluene/MeOH (5 mL/1 mL) were added to a 50 mL three-necked flask under nitrogen. After adding the corresponding 2-bromo-7-iodo-9,9-dimethyl-9H-fluorene (879 mg, 2.2 mmol), the resulting solution was heated to 85 °C for 8 h. After cooling to room temperature, the reaction mixture was diluted with diethyl ether and the organic phase was washed with brine. After drying over $MgSO_4$, the solvent was removed. The resulting crude product was passed through a flash column chromatograph using DCM: petroleum ether mixture (1:10, v/v) as eluent to remove impurities followed by recrystallization from ethanol to obtain a green product in a yield of 83%. ^{1}H NMR (500 MHz, $CDCl_3$), δ(ppm): 8.10 – 8.03 (m, 4H), 7.96 – 7.90 (m, 4H), 7.72 (d, J = 8.0 Hz, 2H), 7.67 (d, J = 1.8 Hz, 2H), 7.57 (dd, J = 8.0, 1.8 Hz, 2H), 1.64 (s, 12H). ^{13}C

NMR (126 MHz, $CDCl_3$), δ(ppm): 156.14, 154.23, 153.67, 138.47, 137.82, 136.88, 133.52, 130.30, 128.64, 128.07, 126.28, 123.68, 121.68, 121.43, 120.21, 47.37, 29.73, 27.12. HRMS (m/z) calcd. for C36H26Br2N2S $[M + H]^+$: 676.0261; found: 677.0262. Anal. calcd. for C36H26Br2N2S (%): C, 63.73; H, 3.86; N, 4.13; S, 4.73. found: C, 63.72; H, 3.85; N, 4.14; S, 4.73.

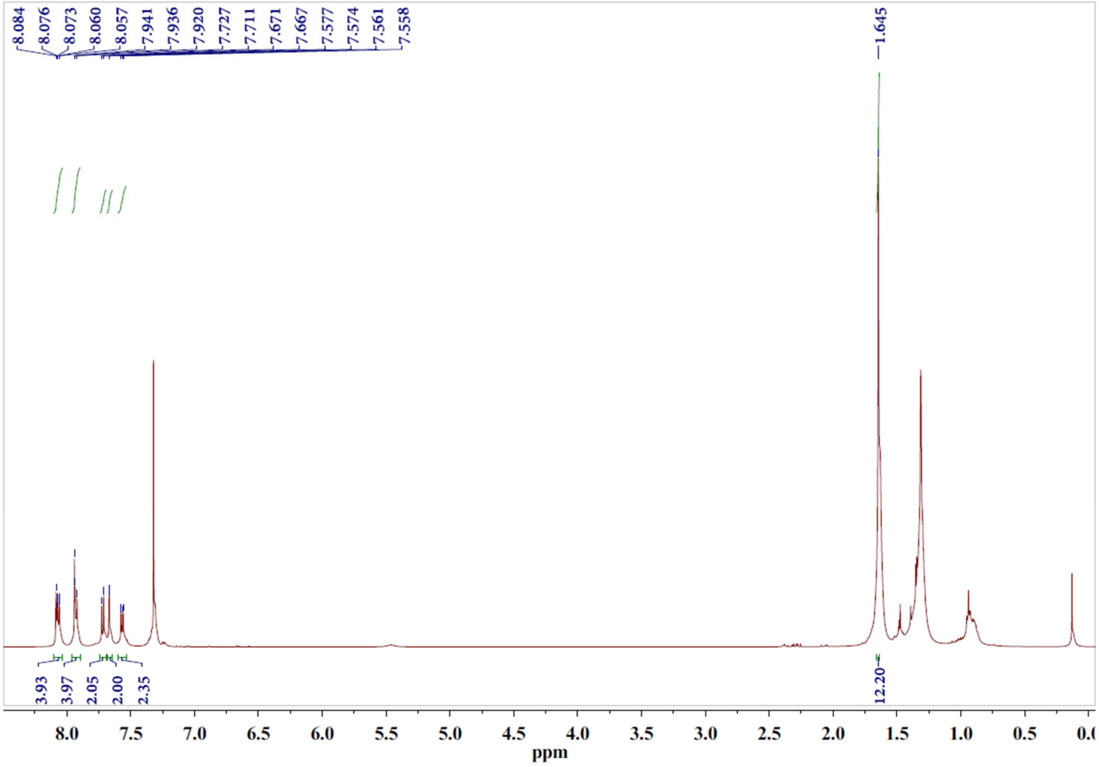

Supplementary Fig. 2. ^{1}H NMR spectrum of BTD-FLBr in $CDCl_3$.

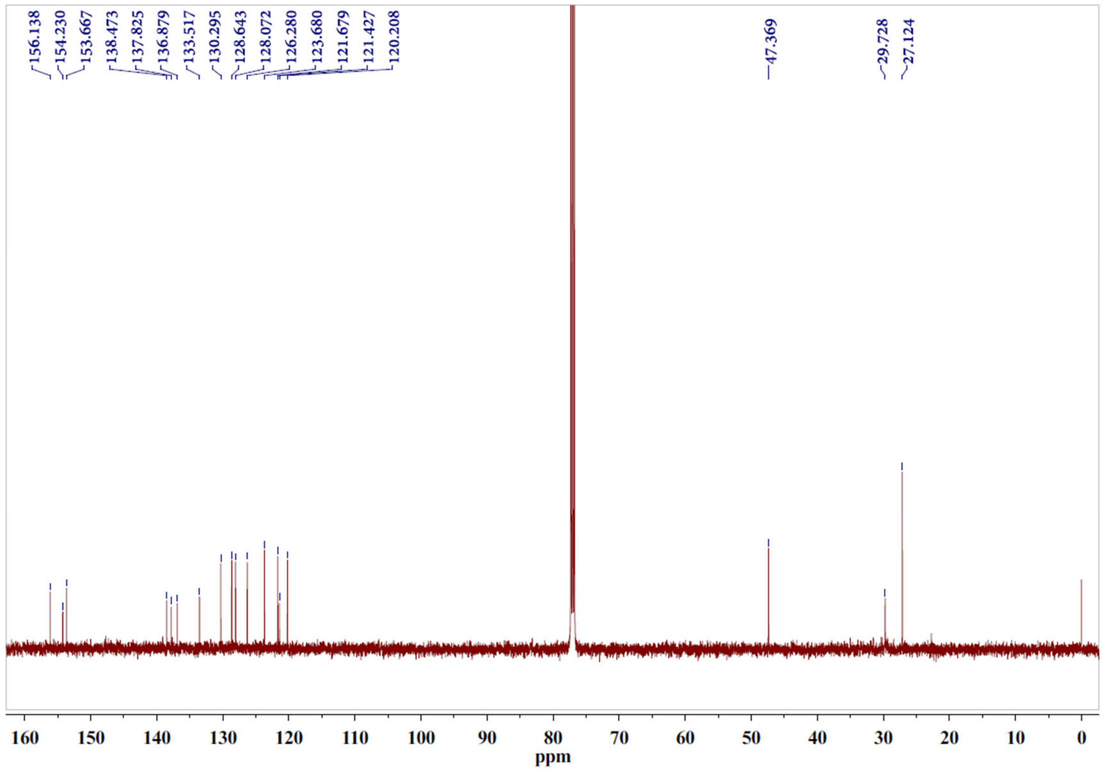

Supplementary Fig. 3. ^{13}C NMR spectrum of BTD-FLBr in $CDCl_3$**.**

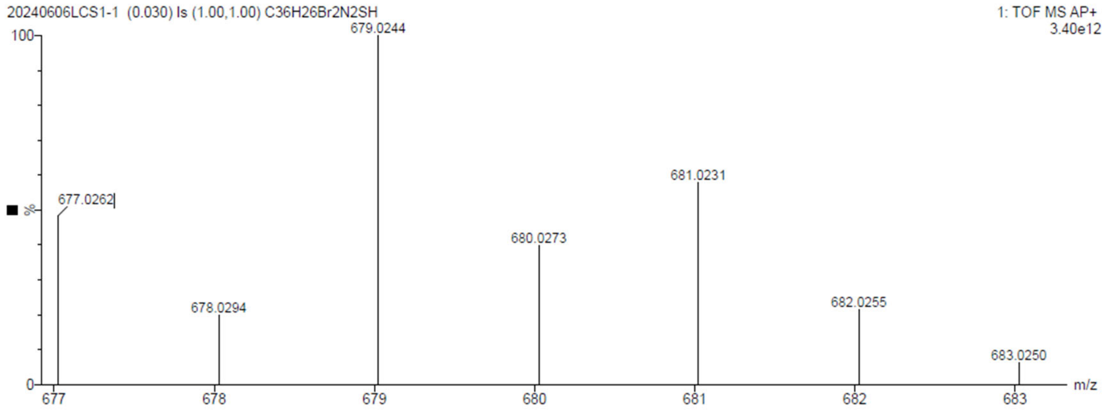

Supplementary Fig. 4. HR-MS spectrum of BTD-FLBr.

Synthesis of BTD-HeBr: 4,7-Bis(4,4,5,5-tetramethyl-1,3,2-dioxaborolan-2-yl)benzo[c][1,2,5]thiadiazole (388 mg 1 mmol), tetrakis-(triphenylphosphine)palladium(0) (16 mg, 0.0144 mol), 2 M K_2CO_3 (6 mL) and toluene/MeOH (5 mL/1 mL) were added to a 50 mL three-necked flask under nitrogen. After adding the corresponding 2-bromo-9,9-bis(6-bromohexyl)-9H-fluorene (1257 mg, 2.2 mmol), the resulting solution was heated to 85 °C for 8 h. After cooling to room temperature, the reaction mixture was diluted with diethyl ether and the organic phase

was washed with brine. After drying over $MgSO_4$, the solvent was removed. The resulting crude product was passed through a flash column chromatograph using DCM: petroleum ether mixture (1:10, v/v) as eluent to remove impurities followed by recrystallization from ethanol to obtain a green product in a yield of 85%. ^{1}H NMR (500 MHz, $CDCl_3$), δ(ppm): 8.05 (d, J = 7.8 Hz, 1H), 8.01 (s, 1H), 7.99 – 7.89 (m, 2H), 7.82 (s, 1H), 7.41 (dd, J = 10.4, 4.7 Hz, 3H), 3.31 (t, J = 6.8 Hz, 4H), 2.22 – 1.99 (m, 4H), 1.79 – 1.67 (m, 4H), 1.58 (s, 3H), 1.16 (dd, J = 14.7, 7.3 Hz, 3H), 0.84 (dd, J = 30.6, 24.1 Hz, 5H). ^{13}C NMR (126 MHz, $CDCl_3$), δ(ppm): δ 154.39, 150.97, 150.76, 141.38, 140.72, 136.32, 133.55, 128.30, 128.03, 127.45, 127.08, 123.91, 122.93, 120.10, 119.87, 55.16, 40.19, 34.08, 32.67, 29.12, 27.79, 23.69. HRMS (m/z) calcd. for C56H64Br4N2S $[M + H]^+$: 1135.1422; found: 1112.1421. Anal. calcd for C56H64Br4N2S (%): C, 60.23; H, 5.78; N, 2.51; S, 2.87. found: C, 60.25; H, 5.76; N, 2.50; S, 2.88.

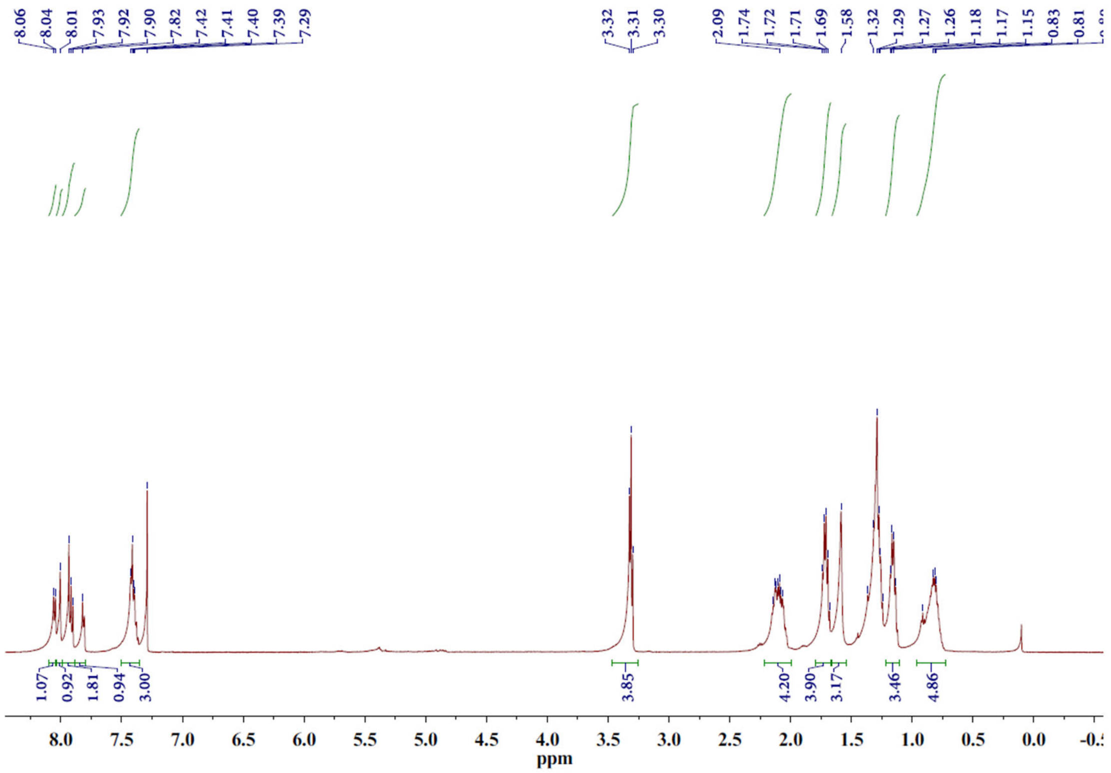

Supplementary Fig. 5. ^{1}H NMR spectrum of BTD-HeBr in $CDCl_3$.

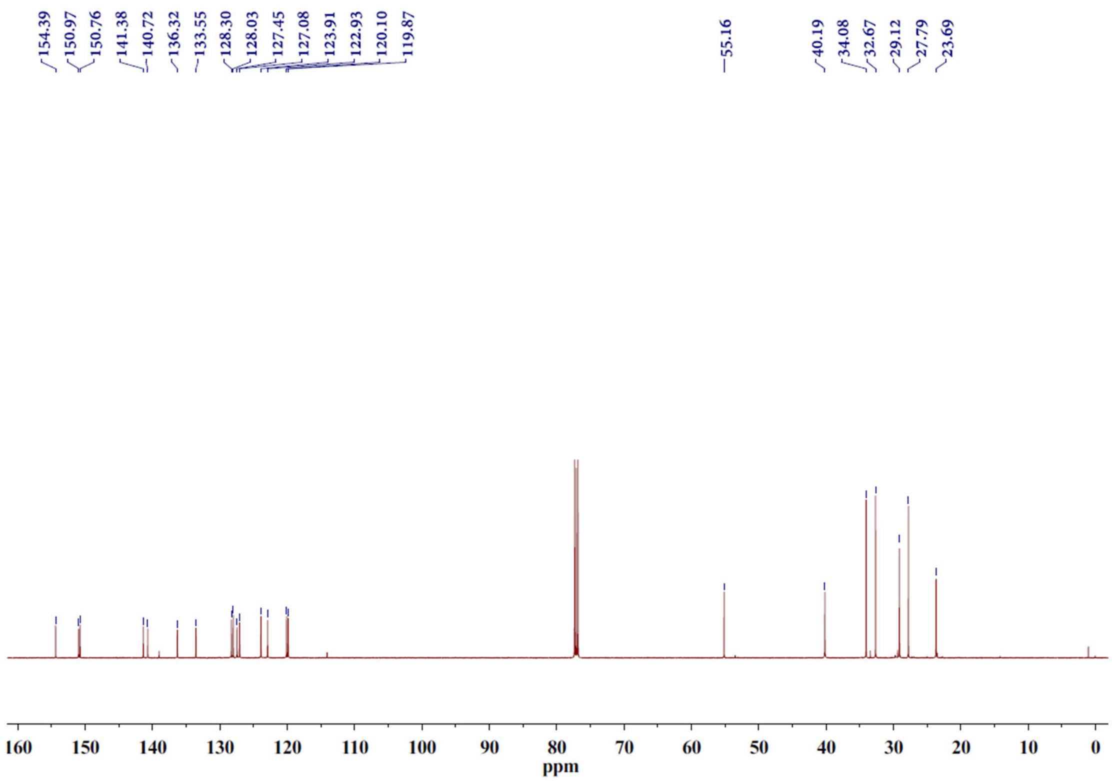

Supplementary Fig. 6. ^{13}C NMR spectrum of BTD-HeBr in $CDCl_3$.

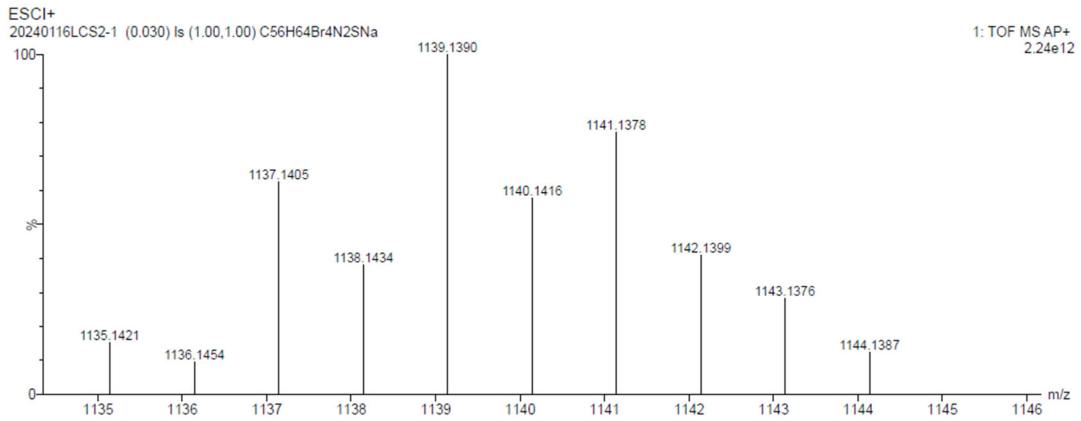

Supplementary Fig. 7. HR-MS spectrum of BTD-HeBr in $CDCl_3$.

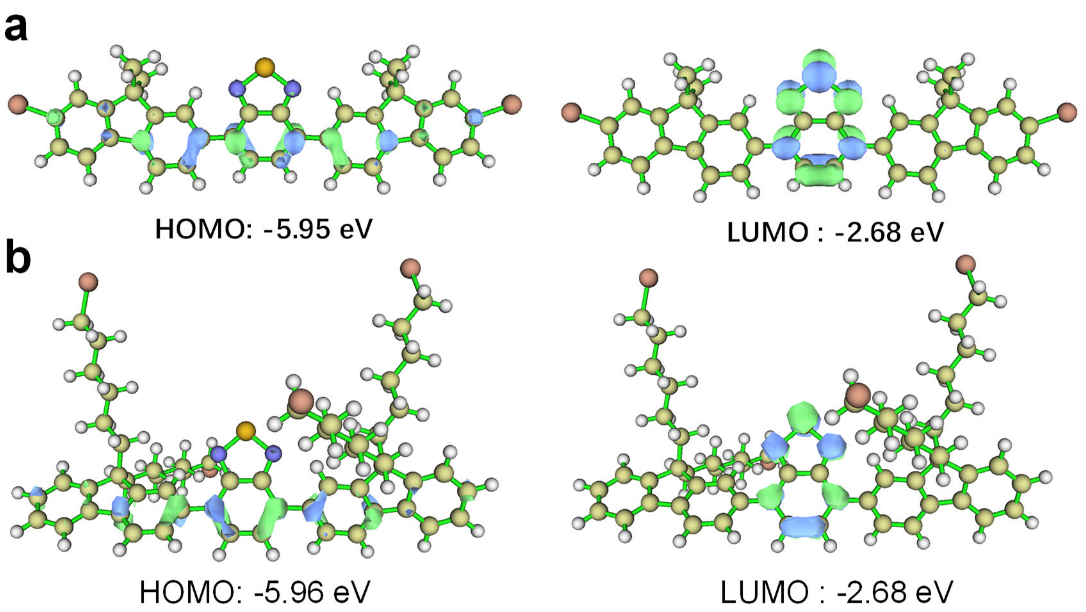

Supplementary Fig. 8. Frontier orbital distributions of (a) BTD-FLBr and (b) BTD-HeBr.

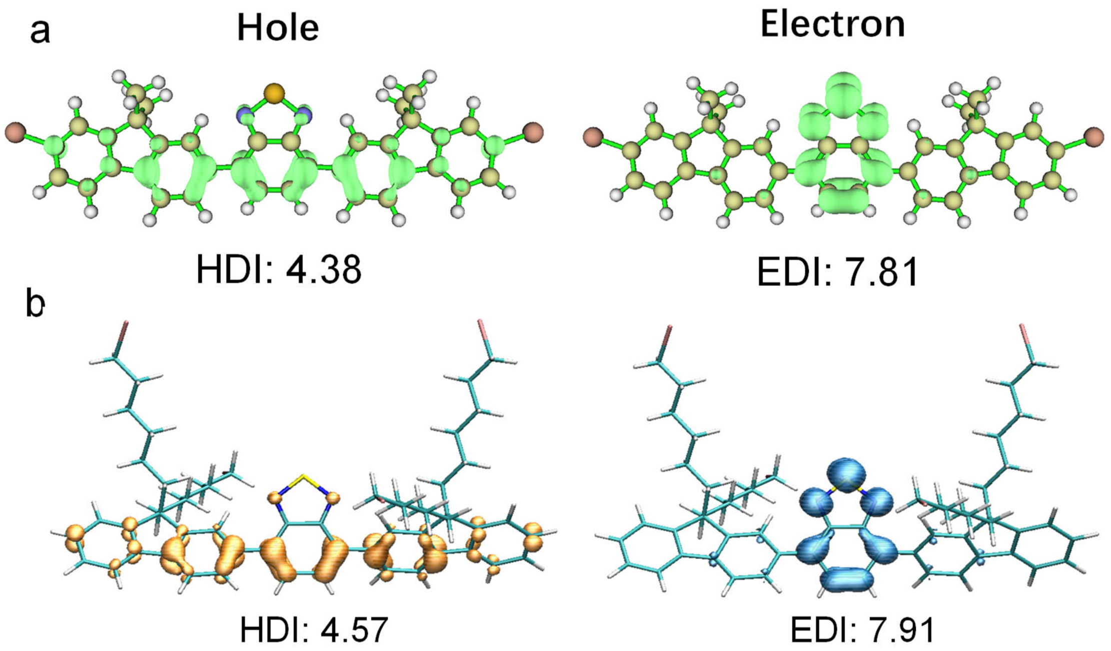

Supplementary Fig. 9. Natural transition orbital analysis and distributions of electron and hole wave functions of S_1 for (a) BTD-FLBr and (b) BTD-HeBr. HDI: hole delocalization index; EDI: electron delocalization index.

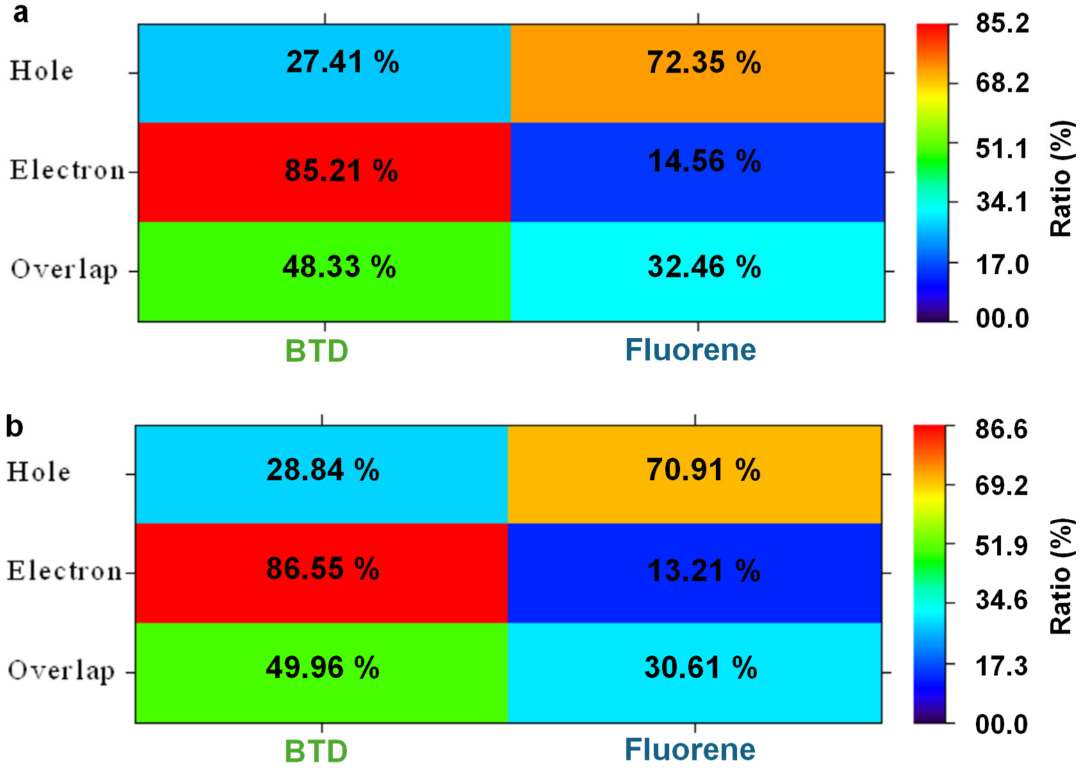

Supplementary Fig. 10. The heat maps of hole, electron, and overlap wave functions in BTD and fluorene fragments of S_1 for (a) BTD-FLBr and (b) BTD-HeBr. The percentages are electron redistribution ratios of the two fragments.

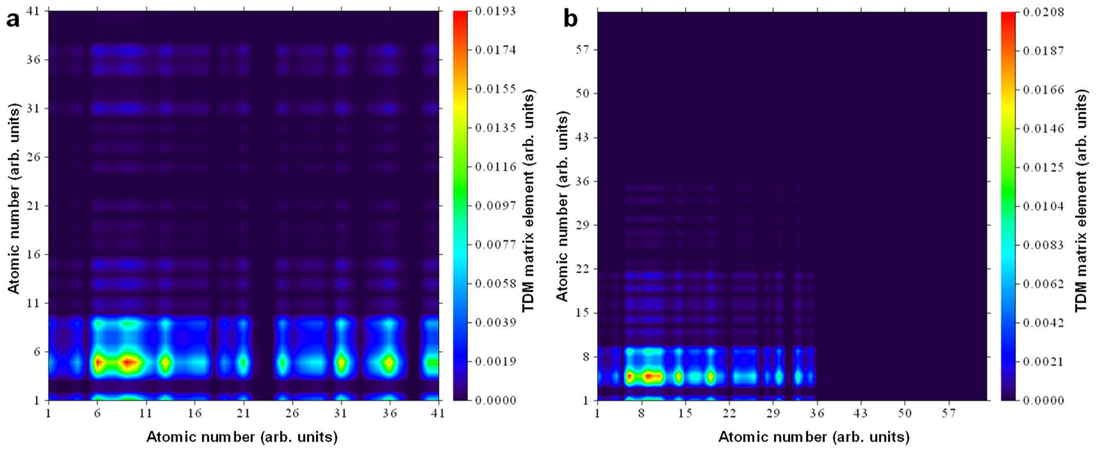

Supplementary Fig. 11. Color-filled transition density matrix (TDM) maps for (a) BTD-FLBr and (b) BTD-HeBr. The No.1 to 9 atoms belong to the BTD fragment and the No.10 to 41 atoms belong to fluorene fragment with bromine atoms for BTD-FLBr. The No.1 to 9 atoms belong to the BTD fragment and the No.10 to 35 atoms belong to fluorene fragment without alkyl bromides for BTD-HeBr.

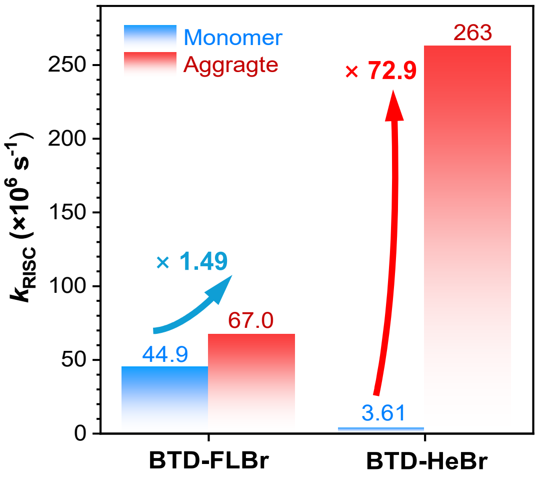

Supplementary Fig. 12. The rate of RISC for BTD-FLBr and BTD-HeBr molecules in monomer and aggregation state.

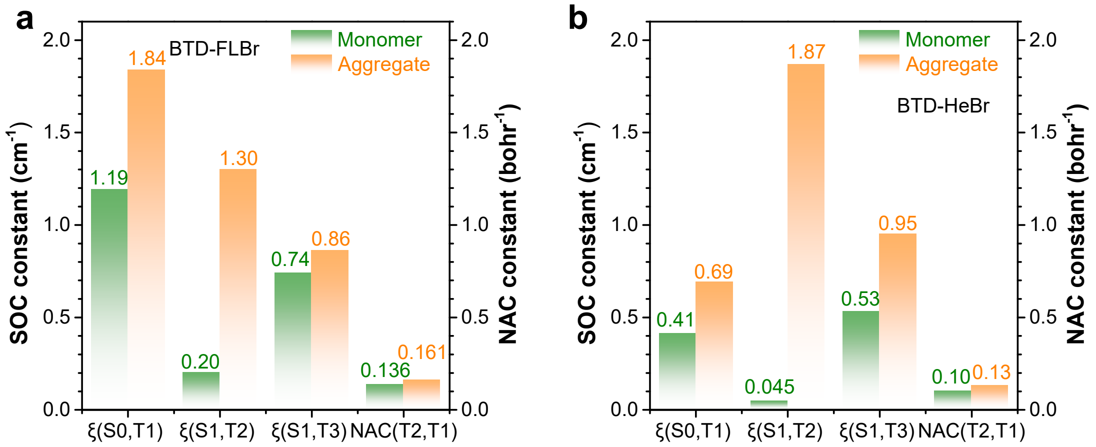

Supplementary Fig. 13. SOC and NAC constants for (a) BTD-FLBr and (b) BTD-HeBr molecules in monomer and aggregation state.

The rate of RISC process between T_n and S_1 states (k_{RISC}) can be computed using the semiclassical Marcus theory and the Fermi golden rule as follows:

$$k_{\mathrm{RISC}} = \frac{\langle S_1|H_{\mathrm{SOC}}|T_n\rangle^2}{\hbar}\sqrt{\frac{\pi}{\Lambda k_{\mathrm{B}}T}}\exp[-\frac{(\Delta E_{\mathrm{ST}}+\Lambda)^2}{4\Lambda k_{\mathrm{B}}T}] \quad (1)$$

where k_B is the Boltzmann constant, $\hbar$ is the reduced Planck constant, and T is the temperature. $\langle S_1|H_{SOC}|T_n\rangle$ is the SOC matrix element between S_1 and the involved T_n

manifold. ΔE_{ST} is the involved singlet-triplet energy gap. Λ denotes the reorganization energy associated with the RISC process from the involved T_n state to S_1. Because the two involved excited states have similar energies and structures, Λ can be quite small and comparable. Thus, Λ is assumed to have a reasonable value of 0.10 eV for the investigated structurally similar molecules.

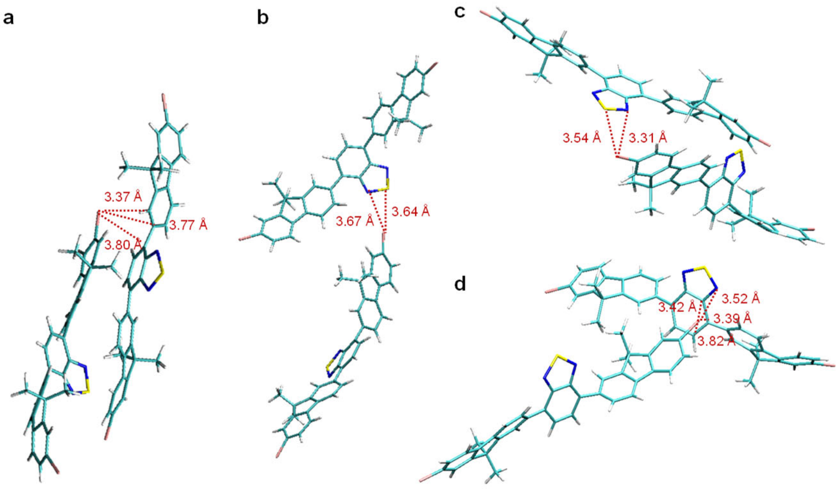

Supplementary Fig. 14. Simulated aggregated state configuration of BTD-FLBr. The aggregate environment and the intermolecular interactions of BTD-FLBr was simulated using the ONIOM model with a combined quantum mechanics and molecular mechanics (QM/MM) approach. The central molecule acts as the high layer with QM at the B3LYP-D3/6-31G(d,p) level, while the surrounding molecules acts as the low layer using the universal force field (UFF).

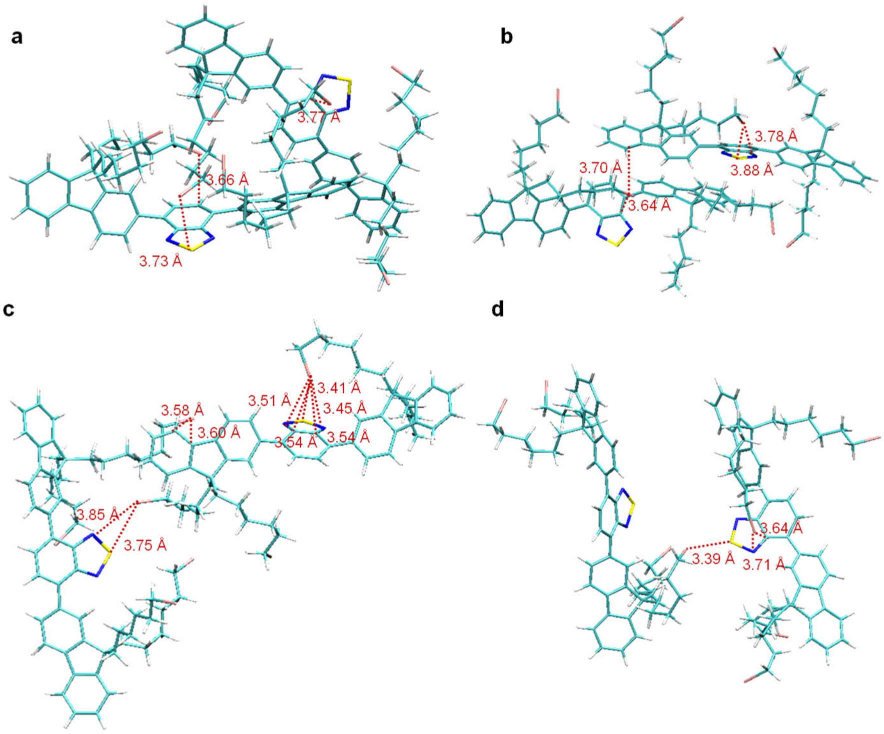

Supplementary Fig. 15. Simulated aggregated state configuration of BTD-HeBr. The aggregate environment and the intermolecular interactions of BTD-HeBr was simulated using the ONIOM model with a combined quantum mechanics and molecular mechanics (QM/MM) approach. The central molecule acts as the high layer with QM at the B3LYP-D3/6-31G(d,p) level, while the surrounding molecules acts as the low layer using the universal force field (UFF).

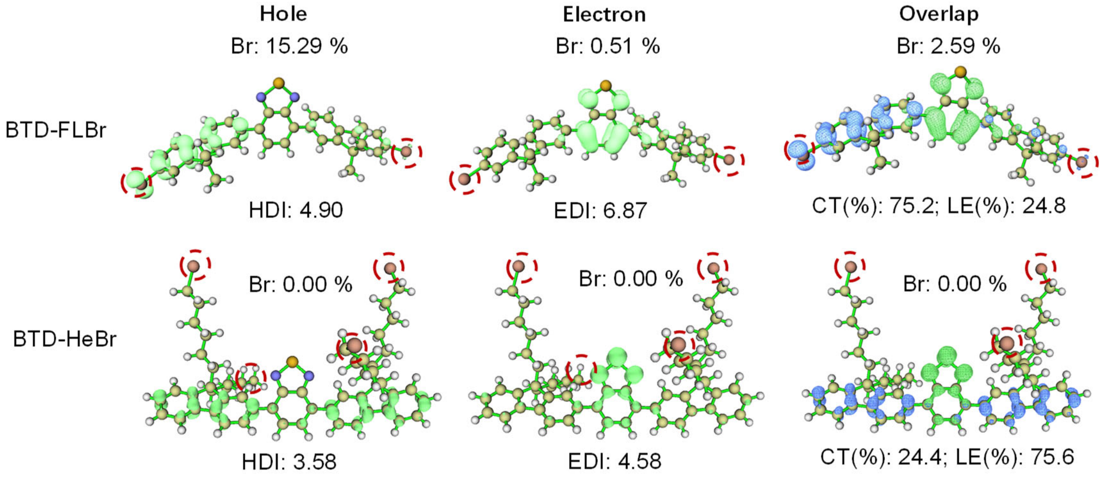

Supplementary Fig. 16. Natural transition orbital analysis and distributions of electron and hole wave functions of T_2 for BTD-FLBr and BTD-HeBr.

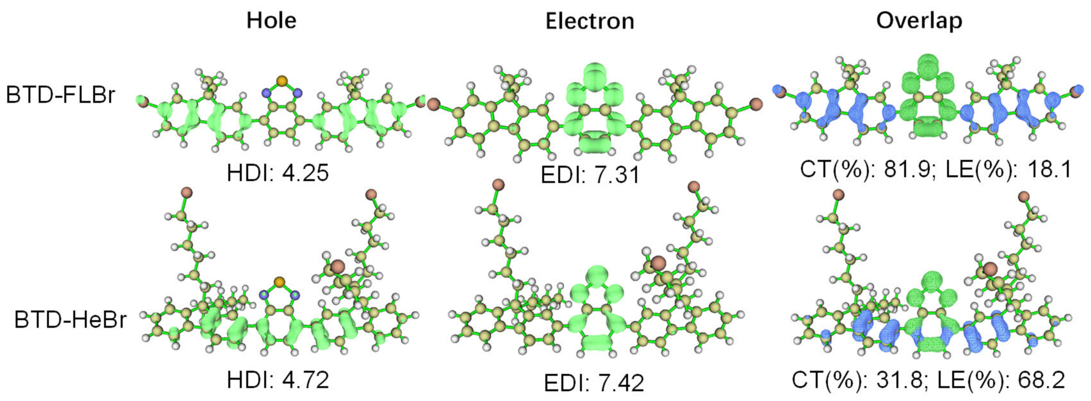

Supplementary Fig. 17. Natural transition orbital analysis and distributions of electron and hole wave functions of T_1 for BTD-FLBr and BTD-HeBr.

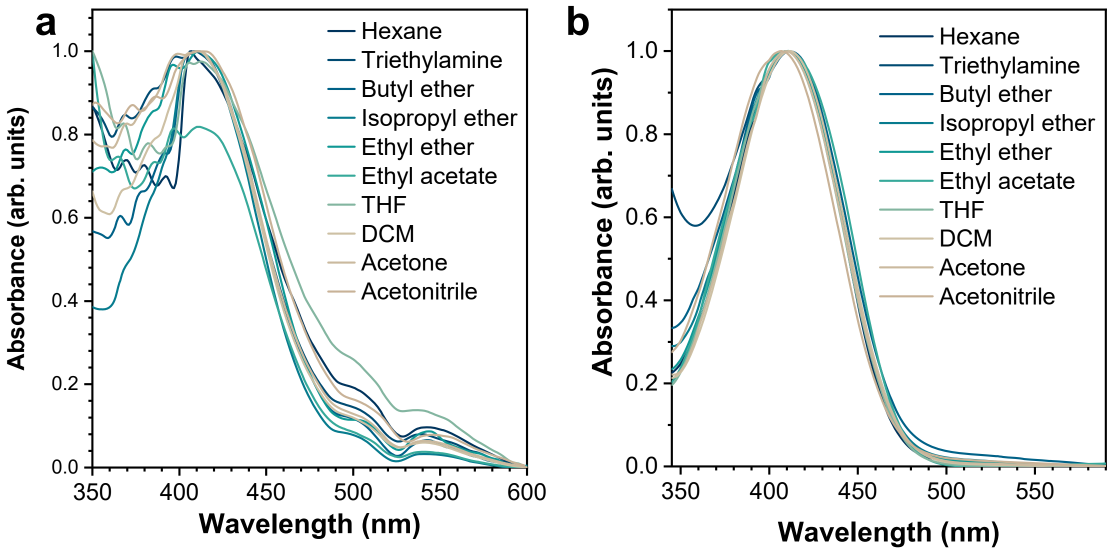

Supplementary Fig. 18. UV-vis spectra of (a) BTD-FLBr and (b) BTD-HeBr in different solvents.

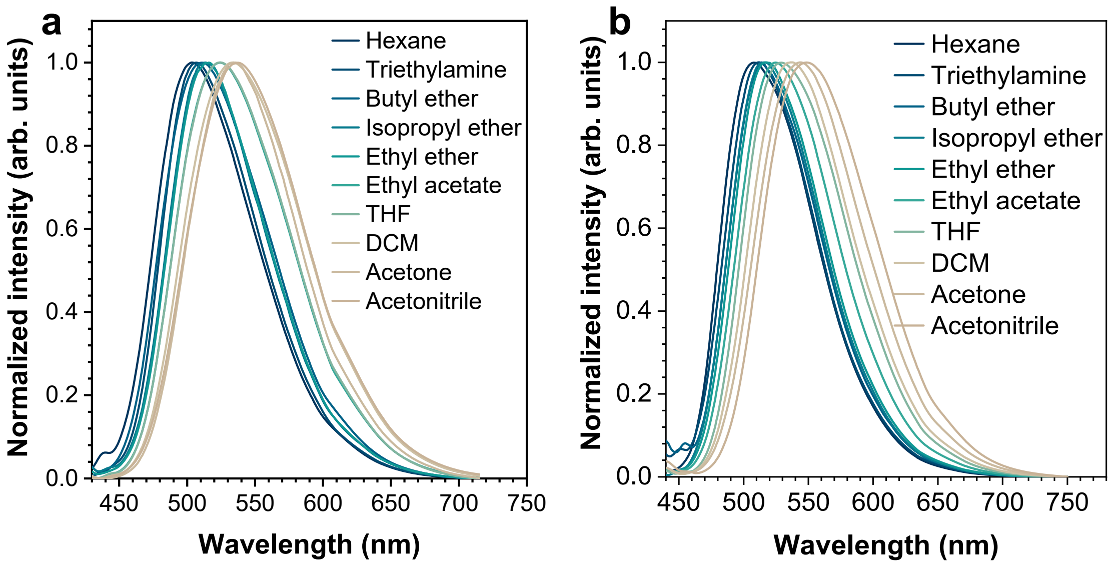

Supplementary Fig. 19. PL spectra of (a) BTD-FLBr and (b) BTD-HeBr in different solvents.

Supplementary Table 1. Detailed absorption and emission peak positions of BTD-FLBr and BTD-HeBr in different solvents.

		BTD-FLBr			BTD-HeBr		
**Solvent**	**$f(\varepsilon,n)$**	**λ_a (nm)**	**λ_f (nm)**	**ν_a-ν_f (cm^{-1})**	**λ_a (nm)**	**λ_f (nm)**	**ν_a-ν_f (cm^{-1})**
Hexane	0.0012	407	504	4729	410	508	4705
Triethylamine	0.048	409	507	4726	410	512	4859
Butyl ether	0.096	411	512	4800	411	514	4876
Isopropyl ether	0.145	410	515	4973	410	516	5010
Ethyl ether	0.167	411	517	4989	410	518	5085
Ethyl acetate	0.2	410	525	5343	409	525	5402
Tetrahydrofuran	0.21	411	531	5499	410	529	5522
Dichloromethane	0.217	411	537	5709	411	537	5708
Acetone	0.284	410	542	5940	410	544	6008
Acetonitrile	0.305	411	546	6016	407	548	6322

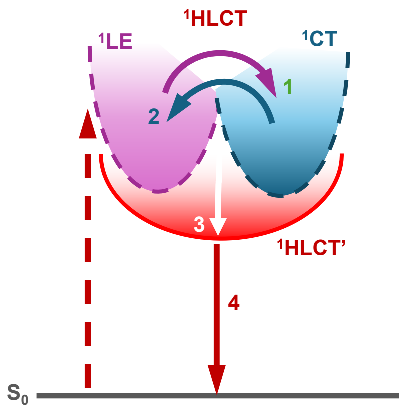

Supplementary Fig. 20. Proposed excited-state deactivation mechanism of BTD-FLBr and BTD-HeBr.

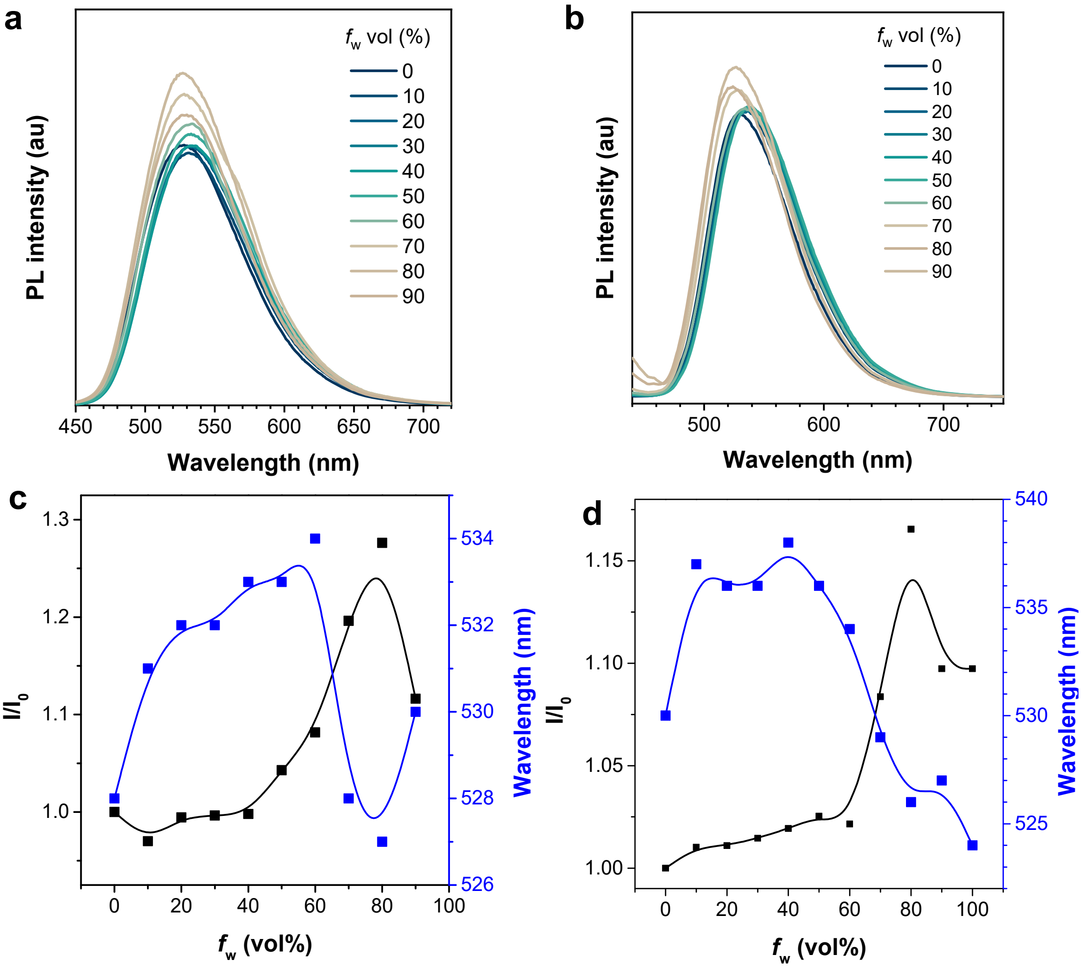

Supplementary Fig. 21. Photoluminescence (PL) properties of BTD-FLBr and BTD-HeBr in H_2O/THF mixtures. The PL spectra of (a) BTD-FLBr and (b) BTD-HeBr and the plot of PL intensity and wavelength of (c) BTD-FLBr, and (d) BTD-HeBr in

H_2O/THF mixtures with different water fractions (f_w) (10^{-5} M). In pure THF, green emission peaked at 523 nm are observed in HLCT emitters. With an increase of f_w, the PL intensity gradually decreases owing to the TICT-dominated luminescent process. When f_w increase from 60% to 80%, the PL intensity reaches maximum accompanied with a blue-shifted in the PL maximum, which is attributed to formation of nanoaggregates to restrain molecular motions. As f_w further increases to 90%, the PL intensity drops sightly due to the formation of small nanosized aggregates with larger specific surface areas, causing a slight quenching of fluorescence.

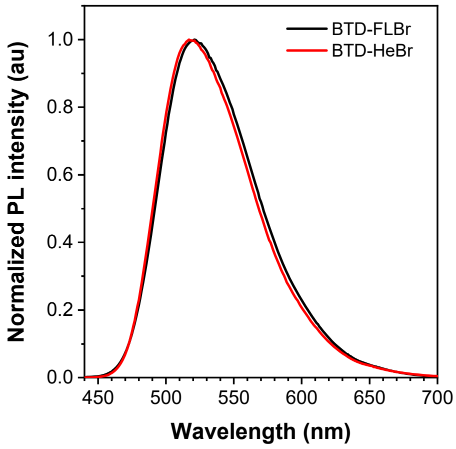

Supplementary Fig. 22. PL spectra of BTD-FLBr and BTD-HeBr in neat films.

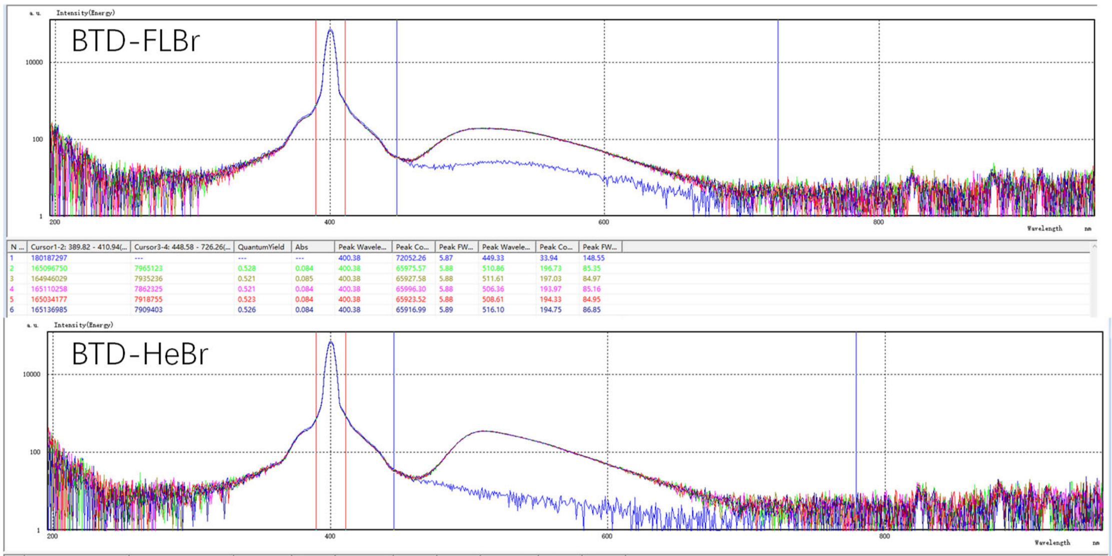

N ...	Cursor1-2: 389.82 - 410.94(...	Cursor3-4: 448.58 - 726.26(...	QuantumYield	Abs	Peak Wavele...	Peak Co...	Peak FW...	Peak Wavele...	Peak Co...	Peak FW...
1	180187297	---	---	---	400.38	72052.26	5.87	449.33	33.94	148.55
2	165096750	7965123	0.528	0.084	400.38	65975.57	5.88	510.86	196.73	85.35
3	164946029	7935236	0.521	0.085	400.38	65927.58	5.88	511.61	197.03	84.97
4	165110258	7862325	0.521	0.084	400.38	65996.30	5.88	506.36	193.97	85.16
5	165034177	7918755	0.523	0.084	400.38	65923.52	5.88	508.61	194.33	84.95
6	165136985	7909403	0.526	0.084	400.38	65916.99	5.89	516.10	194.75	86.85

N ...	Cursor1-2: 389.82 - 410.94(...	Cursor3-4: 445.57 - 779.17(...	QuantumYield	Abs	Peak Wavele...	Peak Co...	Peak FW...	Peak Wavele...	Peak Co...	Peak FW...
1	172702567	---	---	---	400.38	69026.76	5.88	445.57	37.21	121.96
2	161372881	12164012	1.074	0.066	400.38	64576.66	5.87	510.11	352.15	60.09
3	161404881	12147498	1.075	0.065	400.38	64586.35	5.87	512.35	355.12	60.16
4	161356624	12189738	1.074	0.066	400.38	64534.75	5.87	510.11	354.93	59.91
5	161406064	12184473	1.079	0.065	400.38	64683.92	5.87	509.36	354.74	60.47
6	161398911	12143891	1.074	0.065	400.38	64636.60	5.87	512.35	351.14	60.51

Supplementary Fig. 23. PLQYs of BTD-FLBr and BTD-HeBr in neat films.

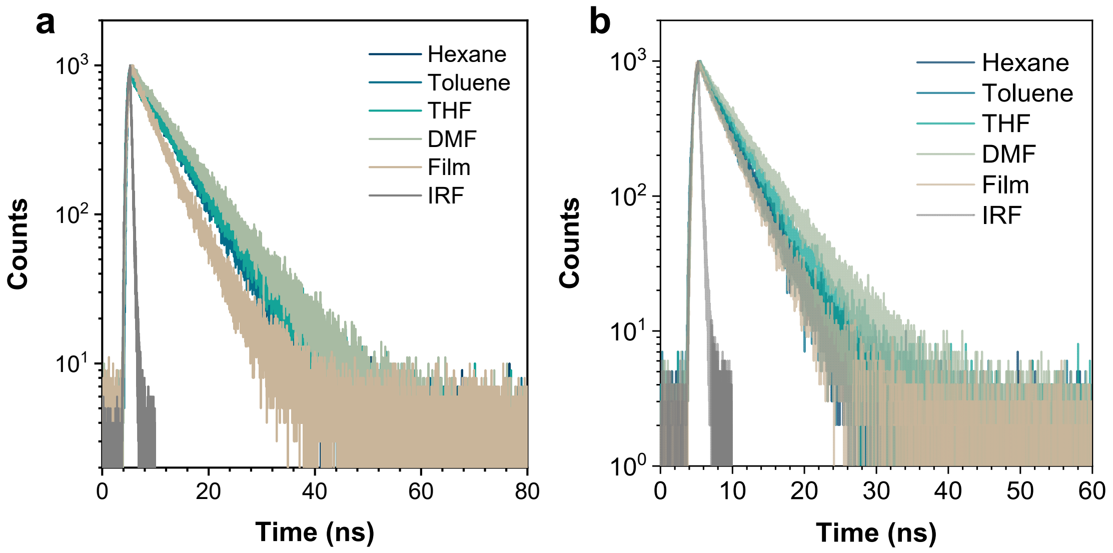

Supplementary Fig. 24. PL decay curves of (a) BTD-FLBr and (b) BTD-HeBr.

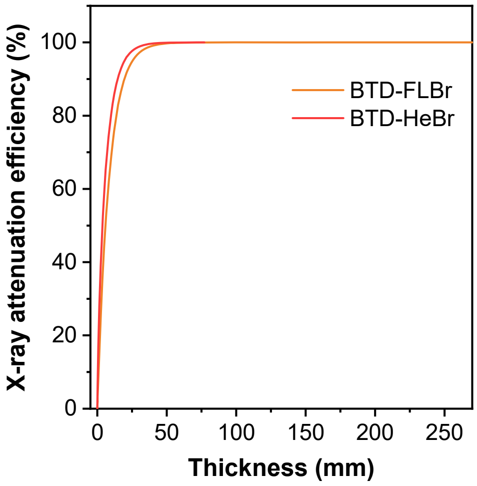

Supplementary Fig. 25. Calculated X-ray attenuation efficiency of BTD-FLBr and BTD-HeBr versus thickness for the entire range of X-ray photon energy (3–50 keV).

The light yield (LY) of the samples was quantified by analyzing the pulse height spectra obtained under excitation with standard ^{241}Am (59.5 keV) γ-ray sources. To address the substantial photon loss caused by the weak penetration of ^{241}Am-derived α particles, which prevents light shield installation, we utilized black aluminum foil to absorb the α particles. The electrical signals generated by the photomultiplier tube (PMT) were collected and digitized using a dedicated data acquisition system. Subsequently, the light yield value was derived by determining the position of the full-

energy peak in the spectrum and conducting a normalization comparison between this peak and the single-photon response of the PMT system. The value can be calculated using the following relationship:

$$\mathrm{LY} = \frac{\mathrm{Bin} \times K}{E \times \eta} \tag{2}$$

where Bin is the channel number corresponding to the full-energy peak; K is the system-related correction parameter (0.043066), including the channel number corresponding to a single photon, amplification factor, single-photon correction, and other items; E is the energy corresponding to the full-energy peak (unit: MeV); and η is the collection efficiency of the photomultiplier tube for the fluorescence band of the samples.

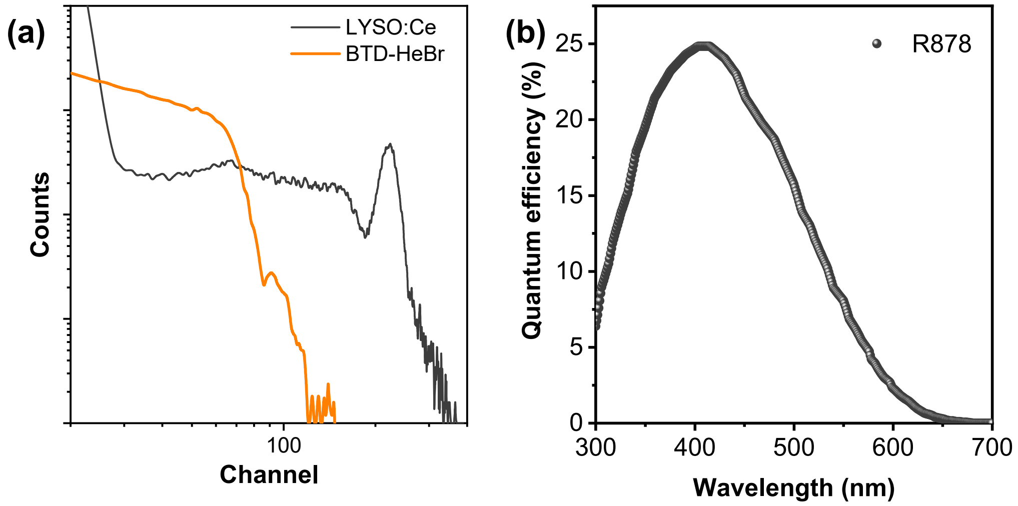

Supplementary Fig. 26. The light yield (LY) of BTD-HeBr quantified by analyzing the pulse height spectra. (a) Pulse height spectrum of BTD-HeBr and LYSO:Ce under ^{241}Am excitation. (b) The quantum efficiency of PMT.

As shown in Supplementary Fig. 26, the two samples present a clear photopeak of the pulse height spectra and the channel numbers are 91 and 224, respectively. The emission-weight quantum efficiency (EWQE) of the PMT used in the characterization is 23.10%. For the collection efficiency of the PMT, the average fluorescence efficiency η=10.35% was obtained via convolution calculation of the radioluminescence (RL)

spectrum of the sample and the fluorescence collection efficiency of the PMT. Therefore, LYSO:Ce has a light yield of 33,000 ± 1,500 photons/ MeV, which is very close to the references confirming the satisfactory accuracy of the gamma-spectrometer and the described method. Since the channel number is linearly proportional to the light yield under the identical test conditions (same K, E, and η), the light yield of BTD-HeBr was derived as approximately 13,400 ± 600 photons/MeV. The error bars were determined from the standard deviation of three independent measurements and the uncertainty of PMT quantum efficiency calibration. This value is higher than that of most organic scintillators, such as EJ200 (10,000 photons/MeV).

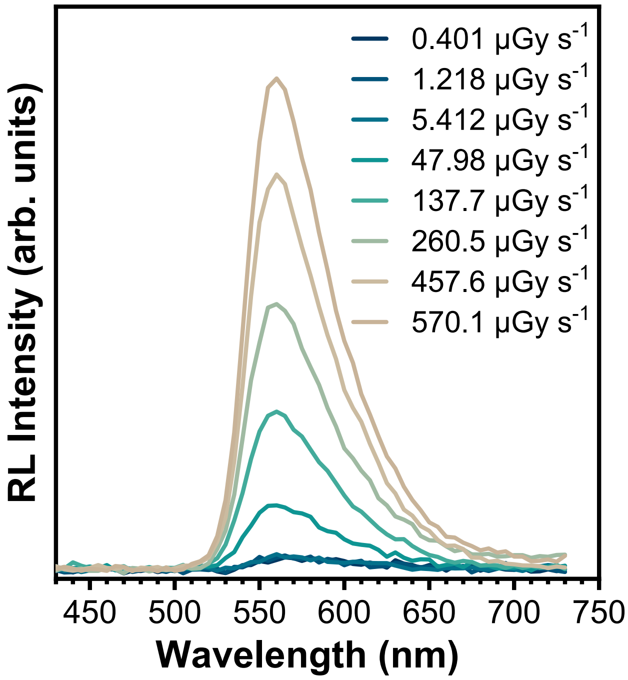

Supplementary Fig. 27. The RL spectra of BTD-HeBr measured at different X-ray dose rate ranging from 0.401 to 570.1 μGy s^{-1}.

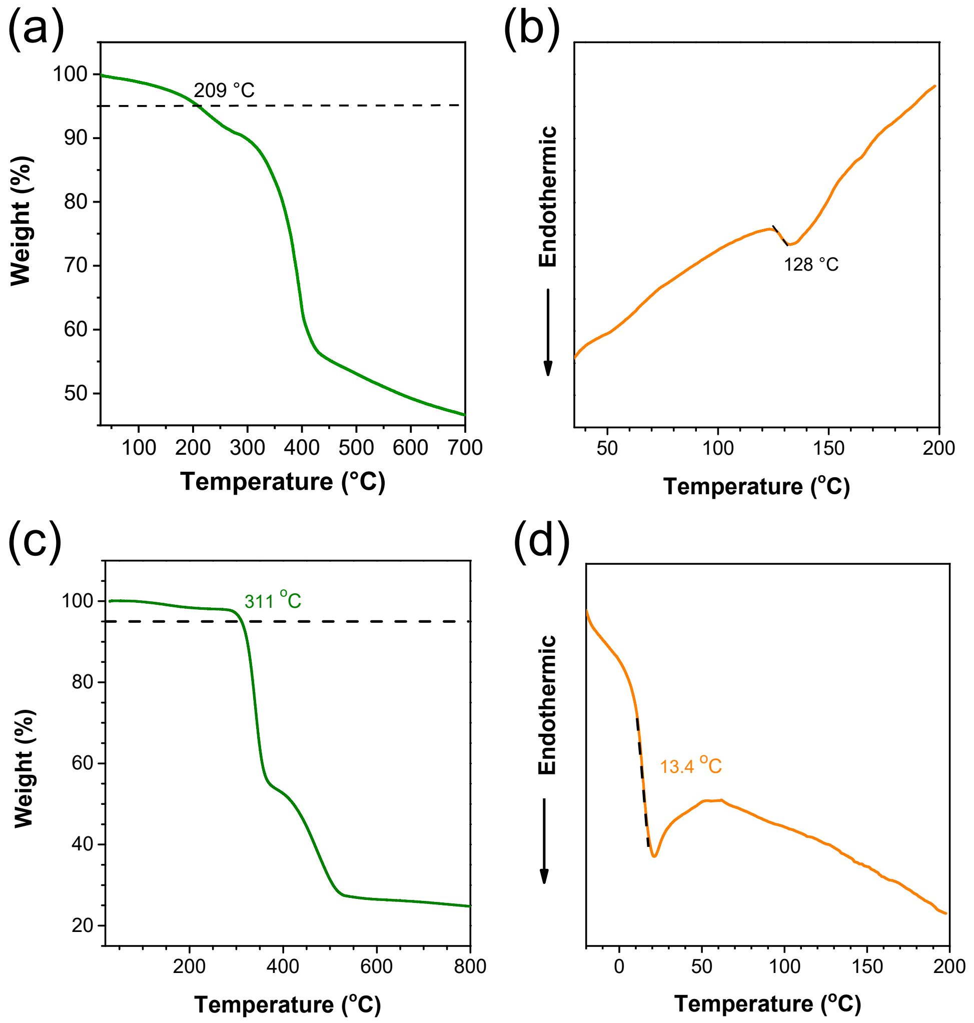

Supplementary Fig. 28. Thermodynamic properties of scintillators. DSC and TGA curves of BTD-FLBr (a,b) and BTD-HeBr (c,d) recorded at a heating rate of 10°C min^{-1}.

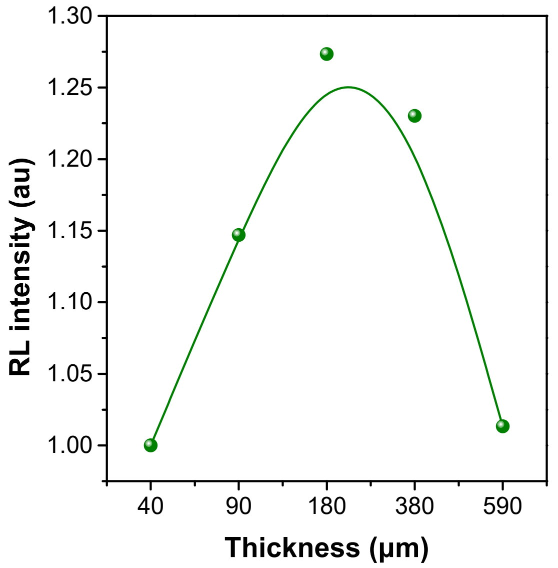

Supplementary Fig. 29. The RL intensity of BTD-HeBr at the different thickness (X-ray tube voltage, 50 kV; dose rate, 2.023 mGy s^{-1}).

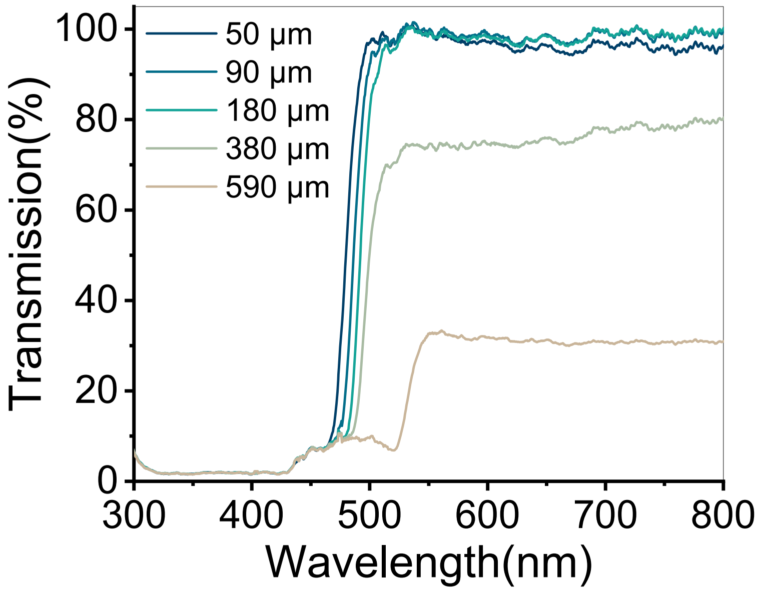

Supplementary Fig. 30. Light transmittance of BTD-HeBr glassy film with different thickness. Film transmittance decreases from 93% (180 μm) to 75% (380 μm) and 33% (590 μm), indicating that the light scattering effect affects the yield of light.

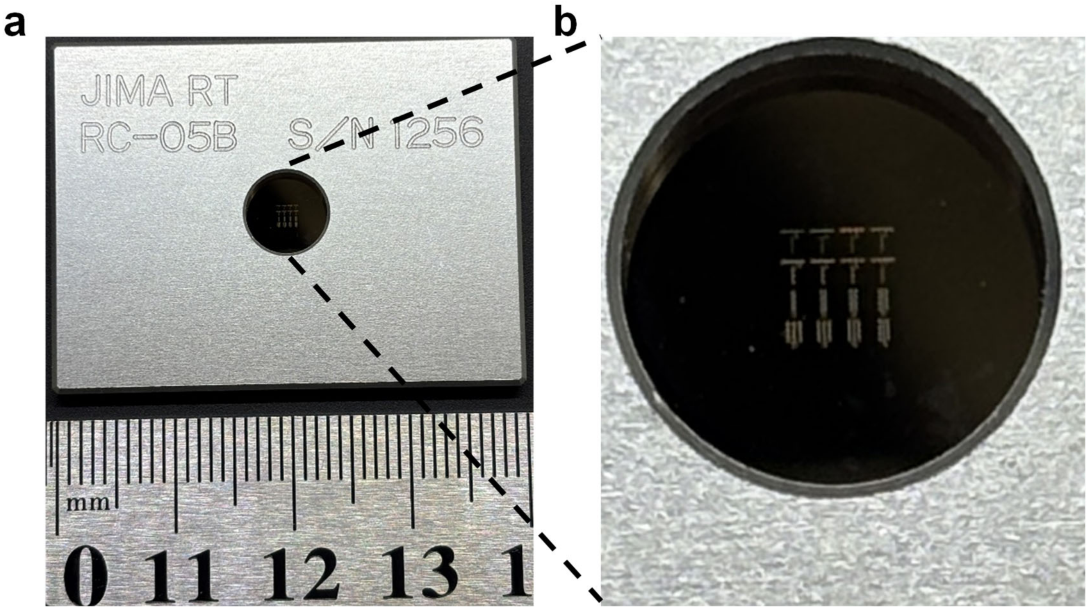

Supplementary Fig. 31. Standard X-ray test pattern plate. (a) Photographs of standard X-ray test pattern plate and the larger image (b).

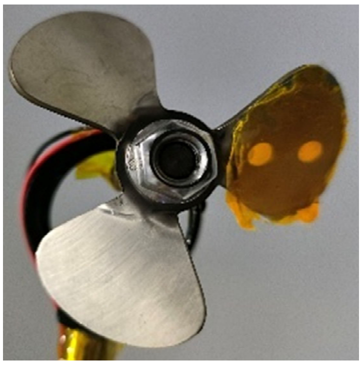

Supplementary Fig. 32. The photograph of fan blades coated with BTD-HeBr film (size: 3.0 × 3.0 cm)**.**

Supplementary Table 2. Comparison of BTD-HeBr scintillator with representative organic scintillators in terms of their spatial resolution and lifetime.

**Number**	**Spatial resolution (lp/mm)**	**Reference**
BTD-HeBr	50.0	This work.
1	16.6	*Nat. Mater.* **21**, 210-216 (2021).
2	18	*Nat. Photon.* **16**, 869-875(2022).

3	18.69	*Nat. Photon.* **18**, 162-169 (2024).
4	20	*Angew. Chem. Int. Ed.* **135**, e202308194 (2023).
5	10	*Angew. Chem. Int. Ed.* **63**, e202402704 (2024).
6	16	*Angew. Chem. Int. Ed.* **63**, e202401833 (2024).
7	15	*Aggregate*, **5**, e485 (2024).
8	25	*Adv. Funct. Mater.* 2400436 (2024).
9	27	*Adv. Opt. Mater.* **12**, 2400113 (2024).
10	9.8	*Adv. Opt. Mater.* **11**, 2202169 (2023).
11	20	*Adv. Mater.*, **33**, 2104749 (2021).
12	8.7	*Adv. Photonics*, **4**, 035002-035002 (2022).
13	16.7	*Small Struct.*, **4**, 2200275 (2023).
14	27.5	*Adv. Sci.*, **10**, 2300406 (2023).
15	12.5	*ACS Cent. Sci.*, **9**, 1419-1426 (2023).
16	5.5	*Chem. Eng. J.*, **430**, 133010 (2022).
17	38.5	*Chem. Sci.* **15**, 18933-18942 (2024).
18	40	*ACS Appl. Mater. Interfaces* **14**, 41275-41282 (2022).

Supplementary References

1. Ma, W. *et al.* Thermally activated delayed fluorescence (TADF) organic molecules for efficient X-ray scintillation and imaging. *Nat. Mater.* **21**, 210-216 (2021).
2. Wang, J.-X. *et al.* Heavy-atom engineering of thermally activated delayed fluorophores for high-performance X-ray imaging scintillators. *Nat. Photon.* **16**, 869-875 (2022).
3. Du, X. *et al.* Efficient and ultrafast organic scintillators by hot exciton manipulation. *Nat. Photon.* **18**, 162-169 (2024).
4. Peng, Q. C. et al. High performance dynamic X-ray flexible imaging realized using a copper iodide cluster-based MOF microcrystal scintillator. *Angew. Chem. Int. Ed.* **135**, e202308194 (2023).
5. Yang, W. et al. Dynamic Reversible Full-Color Piezochromic Fluorogens Featuring Through-Space Charge-Transfer Thermally Activated Delayed Fluorescence and

their Application as X-Ray Imaging Scintillators. *Angew. Chem. Int. Ed.* **63**, e202402704 (2024).
6. Chen, T. et al. Through-space charge-transfer organogold(III) complexes enable high-performance X-ray scintillation and imaging. *Angew. Chem. Int. Ed.* **63**, e202401833 (2024).
7. Zhan, L. *et al.* Organic molecules with dual triplet-harvesting channels enable efficient X-ray scintillation and imaging. *Aggregate*, **5**, e485 (2024).
8. Yuan, S. et al. Thermally Activated Delayed Fluorescent Ag(I) Complexes for Highly Efficient Scintillation and High-Resolution X-Ray Imaging. *Adv. Funct. Mater.* 2400436 (2024).
9. Quan, X. et al. Low-Cost, Large-Area, and Highly Transparent Organic Glassy Scintillators for High Resolution X-Ray Imaging. *Adv. Opt. Mater.* **12**, 2400113 (2024).
10. Liu, X. et al. Thermally Activated Delayed Fluorescent Scintillators Based on Mononuclear Copper(I) Halide Complexes for High-Resolution X-Ray Imaging. *Adv. Opt. Mater.* **11**, 2202169 (2023).
11. Chen, M. *et al.* Organic Semiconductor Single Crystals for X-ray Imaging. *Adv. Mater.* **33**, 2104749 (2021).
12. Wei, J. et al. Organic room-temperature phosphorescent polymers for efficient X-ray scintillation and imaging. *Adv. Photonics*, **4**, 035002-035002 (2022).
13. Sun, Q. et al. Nano Organic Co-Crystal Scintillator for X-ray Imaging. *Small Struct.*, **4**, 2200275 (2023).
14. Wang, J. X. et al. Singlet Fission-Based High-Resolution X-Ray Imaging Scintillation Screens. *Adv. Sci.*, **10**, 2300406 (2023).
15. Peng, Q.-C. et al. Thermally Activated Delayed Fluorescence Coinage Metal Cluster Scintillator. *ACS Cent. Sci.*, **9**, 1419-1426 (2023).
16. Wang, W.-F. et al. Sensitive X-ray detection and imaging by a scintillating Lead(II)-based Metal-Organic framework. *Chem. Eng. J.*, **430**, 133010 (2022).
17. Yang, X. et al. A hot exciton organic glassy scintillator for high-resolution X-ray imaging. *Chem. Sci.* **15**, 18933-18942 (2024).

18. Chen, M. et al. Anthracene Single-Crystal Scintillators for Computer Tomography Scanning. *ACS Appl. Mater. Interfaces* **14**, 41275-41282 (2022).
